# Cattle–compost–soil: The transfer of antibiotic resistance in livestock agriculture

**DOI:** 10.1002/mbo3.1375

**Published:** 2023-08-18

**Authors:** Fadhel Abbas, Phil Thomas, Bianca Cully‐Duse, Nicholas M. Andronicos, Gal Winter

**Affiliations:** ^1^ School of Science and Technology The University of New England Armidale New South Wales Australia

**Keywords:** AMR, antibiotic resistance, antimicrobial resistance, compost, resistome, soil microbiology

## Abstract

Antibiotic resistance is a major global health threat. Agricultural use of antibiotics is considered to be a main contributor to the issue, influencing both animals and humans as defined by the One Health approach. The purpose of the present study was to determine the abundance of antibiotic‐resistant bacterial populations and the overall bacterial diversity of cattle farm soils that have been treated with animal manure compost. Soil and manure samples were collected from different sites at Tullimba farm, NSW. Cultures were grown from these samples in the presence of 11 commonly used antibiotics and antibiotic‐resistant bacteria (ARB) colonies were identified. Soil and manure bacterial diversity was also determined using 16S ribosomal RNA next‐generation sequencing. Results showed that ARB abundance was greatest in fresh manure and significantly lower in composted manure. However, the application of composted manure on paddock soil led to a significant increase in soil ARB abundance. Of the antibiotics tested, the number of ARB in each sample was greatest for antibiotics that inhibited the bacterial cell wall and protein synthesis. Collectively, these results suggest that the transfer of antibiotic resistance from composted animal manure to soil may not be solely mediated through the application of live bacteria and highlight the need for further research into the mechanism of antibiotic resistance transfer.

## Graphical abstract

The study describes the analysis of antibiotic‐resistant bacteria (ARB) abundance and the overall bacterial diversity of cattle farm soils that have been treated with animal manure compost. Our results showed that ARB abundance was greatest in fresh manure and significantly lower in composted manure. However, the application of composted manure on paddock soil led to a significant increase in soil ARB abundance. The paper discusses this apparent conflict and raises hypotheses for future studies to explain it.

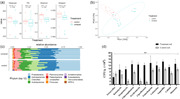

## 1 INTRODUCTION

Antimicrobial resistance, demonstrated by the high abundance of antibiotic‐resistant bacteria (ARB) and antibiotic‐resistance genes (ARGs) in clinical and environmental settings, represents a significant global threat to human and animal health (Udikovic‐Kolic et al., 2014). There is a growing concern surrounding the spread of antibiotic resistance from livestock to humans, mainly due to the prophylactic administration of antibiotics to livestock in large‐scale agricultural settings. For example, approximately 80% of all antibiotics sold in the United States are used as livestock growth promoters and disease prevention. Similarly, in China and Japan, 52% and 50% of the manufactured antibiotics, respectively, are used for growth promotion in animal production (Lekshmi et al., 2017; Zhang et al., 2015). Growth‐promoting antibiotics are given at subtherapeutic doses. They improve feed conversion and animal growth and reduced morbidity and mortality due to clinical and subclinical diseases. The average growth improvement was estimated to be between 4% and 8%, and feed utilization was improved by 2%–5% (Butaye et al., 2003).

One commonly used class of growth promoter antibiotics is ionophores. Ionophores are a diverse class of antibiotics, largely produced by the bacterial genus *Streptomyces* (Badger et al., 2020; Butaye et al., 2003; Wong, 2019). They are deemed as not medically important as they are not used in human medicine. In the United States, 4.6 million kilograms of ionophores were sold in 2016 with this number expected to increase significantly (Wong, 2019). In Australia, cattle farmers reported the ionophore monensin as the most prevalently used in‐feed growth promoter (Badger et al., 2020). Although the underpinning mechanisms are still unknown, ionophores assume to contribute to growth promotion through alterations to ruminal fermentation (Badger et al., 2020; Butaye et al., 2003), presumably due to changes in the gut microbiome. Ionophores may also have direct impacts on the host's metabolism and physiology (Armstrong & Spears, 1988).

The consequence of prolonged antibiotic use in the livestock industry is the widespread increase of antibiotic‐resistant populations of gastrointestinal tract microbial communities in production animals. If livestock are prophylactically treated with the same antibiotics used to treat bacterial infections in human medicine, then a reservoir for the ARB and ARG increase within the human food supply chain (CSIRO, 2023; Sazykin et al., 2021; Tian et al., 2021). Moreover, even the use of antibiotics that are not used in humans, like ionophores, still carry risk, due to the possibility of cross‐resistance or co‐selection (Butaye et al., 2003; Wong, 2019), for example, resistance to monensin was found to be correlated with resistance to the antibiotic vancomycin. Not only does this pose a threat to humans directly consuming animal products, but ARB and ARGs that are released in animal feces are also capable of spreading through the environment (CSIRO, 2023; Lekshmi et al., 2017). It is estimated that up to 95% of the antibiotics fed to animals are excreted in animal manure (Gutiérrez et al., 2010) and that a significant fraction of these excreted compounds maintains their activity within the soil (Gaballah et al., 2021). From a microbial ecology point of view, these excreted antibiotics act as a selective pressure in the microbial community of the soil, reduce the overall microbial diversity of the soil, and enhance the abundance of ARB in the soil. From the soil, antibiotic resistance can spread further into crops intended for human consumption and/or to water reservoirs (Gaballah et al., 2021). This phenomenon is best described by the One Health approach, a conceptual framework that refers to the relationship between the health of humans, animals, and the environment (One Health Commission 2021; McEwen & Collignon, 2018).

In some farming operations, animal manure is stored and composted. During composting, the manure undergoes physical and nutritional profile changes. Hence, the composting process may also affect the microbial populations in the manure and has been shown to reduce the abundance of human pathogens and antibiotic‐resistant microorganisms (Gaballah et al., 2021; Pu et al., 2019; J. Wang et al., 2015; L. Wang et al., 2019). Agricultural soils are treated with animal manure compost at high frequency and thus represent a significant ecological reservoir for ARB and ARGs, containing more than 30% of the known ARGs in public databases (Nesme & Simonet, 2015). Therefore, it is hypothesized that the soil that has been treated with composted manure from feedlot cattle‐fed monensin‐supplemented rations will have lower microbial diversity as well as an increased abundance of ARB compared to uncomposted soil. To this end, bacterial diversity and the prevalence of ARB populations were evaluated in cattle farm soil that was treated with compost compared to control soils. The antibiotic resistance survey included 11 different antibiotics commonly used in agriculture and/or human health.

## 2 MATERIALS AND METHODS

### 2.1 Soil and manure samples collection

**Figure 1:**
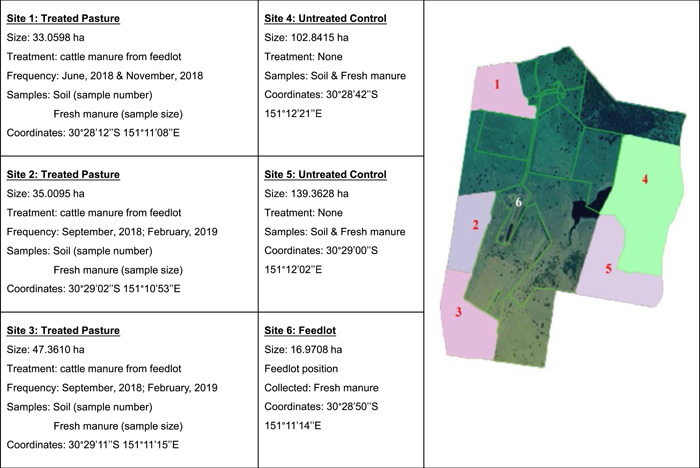
Aerial view of sample collection sites in Tullimba farm of Armidale, NSW, Australia.

Soil and manure samples were collected from the Tullimba farm (University of New England, Armidale, NSW, Australia) located 40 km southwest of Armidale, NSW. The Tullimba farm is spread over 740 ha and has approximately 1000 head of cattle. Carboxylic ionophore (Monensin) is the only antibiotic included in standard cattle diets on the Tullimba farm (Table 1). A total of 500 soil subsamples and 130 manure subsamples were collected from different sites on the Tullimba farm between April and June 2019 (Figure 1).

**Table 1:** Standard diet formulation feed to cattle on the Tullimba feedlot/farm.

Component	DM%	Starter	T1	T2	Finisher
Ration DM	%	75.61	75.38	75.89	74.92
Protein	%	13.80	13.77	13.87	14.22
Eq Prot	%	0.63	0.73	0.90	1.00
ME (MJ/kg)	‐	11.58	11.85	12.11	12.46
NEm	Mcal/kg	1.83	1.89	1.95	2.02
NEg	Mcal/kg	1.20	1.25	1.31	1.38
NDF	%	33.73	30.57	27.00	23.46
eNDF (%DM)	%	20.25	17.79	14.67	11.30
Fat	%	4.52	4.60	4.68	5.09
VitA	IU/kg	1.620.11	1.857.33	2.305.90	2.569.33
VitE	IU/kg	5.55	6.37	7.91	8.81
Calcium	%	0.58	0.63	0.74	0.80
Phosphorus	%	0.48	0.45	0.43	0.43
Monensin	ppm	15.28	17.51	21.74	24.23

Abbreviations: DM, dry matter; Starter, bingeing feed of cattle with low grains; Eq Prot, Equivalent Protein; ME, metabolisable energy; NDF, neutral detergent fibre; NE, Net energy; NEg, net energy gain; T1, first transition (second line to feed cattle (high grain); T2, second transition (third line to feed cattle (higher grain); and finisher (last line of feeding cattle with highest grains); Vit, vitamin.

Soil samples were collected from five paddocks of various sizes (Figure 1). Soil sites 1–3 were chosen due to the regular application of composted cattle manure from the feedlot area every 5 months. Before sampling, site 1 was compost‐treated in June and November 2018. Sites 2 and 3 were treated with compost in September 2018 and February 2019. Sites 4 and 5 had not been treated with composted manure and therefore were selected as control. Three hundred subsamples were collected from treatment sites 1, 2, and 3. While 200 subsamples were collected from control sites 4 and 5. Systematic grid‐square sampling patterns were used to collect soil samples (de Zorzi et al., 2008). Twenty, 22‐mm diameter cores (up to 15 cm deep) were collected from each of the five plots of 50 square meters (100 samples) set up within 4 ha of land in each site.

Manure samples were collected from the different sites. Sixty subsamples of fresh manure (i.e., still soft and warm) were collected from the six sites (paddock and feedlot), each site grazing 50–100 head of cattle. Thirty subsamples of fresh manure from paddock sites were collected and 10 subsamples were combined into a composite sample for testing. Similarly, 30 subsamples of fresh manure from feedlot were also collected and 10 subsamples were combined into a single sample for testing. While 100 manure subsamples were composted for several months onsite by the farm and used for pasture fertilization. Of these composted samples, 33 subsamples were combined into a single sample for analysis. In the lab, these soil and manure samples were kept at 4°C before culturing. Five grams of each sample was placed in a 15‐mL screw cap tube and archived at −80°C for DNA extraction.

### 2.2 Isolation of ARB from field samples

Suspensions of soil and manure samples were made by placing 5 g of soil or manure into a sterile flask with 45 mL of sterile distilled water, shaken at 28°C, 220 rpm for 1 h (Yang et al., 2016) and the slurries were filtered through sterile gauze. The resuspended soil and compost filtrates were serially diluted from 10^−^^1^ to 10^−4^, in sterile distilled water under aseptic conditions.

Diluted manure and soil filtrates (100 µL) containing bacterial populations were cultured on nutrient agar plates (agar 15 g/L, peptone 5.0 g/L, NaCl 5.0 g/L, yeast extract 2.0 g/L, and beef extract 1.0 g/L) (Snyder & Atlas, 2006), each supplemented with a specific antibiotic, tetracycline (256 µg/mL), kanamycin (256 µg/mL), streptomycin (2000 µg/mL), ciprofloxacin (256 µg/mL), trimethoprim (256 µg/mL), cefotaxime (256 µg/mL), chloramphenicol (256 µg/mL), erythromycin (256 µg/mL), ampicillin (256 µg/mL), colistin (256 µg/mL), and monensin (8 µg/mL). Bacteria were defined as ARB if they were able to grow in the presence of the antibiotics used.

### 2.3 Molecular identification of bacterial species isolated from soil and manure

The genomic DNA of ARB isolates was extracted using the Isolate II Genomic DNA Kit (Bioline), according to the manufacturer's instructions. The quantity and concentration of DNA were determined using a Nanodrop 8000c spectrophotometer (Thermofisher Scientific). The bacterial 16S ribosomal RNA (rRNA) gene was amplified using primer pairs (1369‐F, 5′‐CGGTGAATACGTTCYCGG‐3′ and 1541‐R, 5′‐AAGGAGGTGATCCRGCCGCA‐3′) as described previously (Huang et al., 2015). The PCR reaction and amplification program were described in Anisimova & Yarullina (2019). The PCR products were run on a 1% agarose gel and stained with GelRed (Biotium). The PCR product was purified using a QIAquick PCR Purification Kit (Qiagen) according to the manufacturer's instructions and was sequenced at Australian Genome Research Facility using Sanger sequencing technology. Basic Local Alignments Tool (BLAST) algorithm was used to analyze sequence data.

### 2.4 Statistical analysis

Two‐way analysis of variance with Bonferroni post hoc tests was performed on CFU data using Prism version 8.3.1 (GraphPad) to determine statistical significance (i.e., *p* < 0.05).

### 2.5 Analysis of bacterial diversity

Soil microbial DNA was extracted from −80°C archived samples using DNeasy PowerSoil Pro Kit (Qiagen), according to the manufacturer's instructions. Fecal manure was extracted from −80°C archived samples using QIAamp DNA Stool Mini Kit (Qiagen). Microbial profiling of extracted DNA was performed by Illumina MiSeq. The 2 × 300 bp paired‐end sequencing (Ramaciotti Centre for Genomics, Sydney, Australia) of the 16S V3–V4 region of the rRNA gene (primers 341F–805R), from which was obtained a total of 2,948,799 merged sequences. Bacterial 16S rRNA amplicon analysis was performed using QIIME 2 2021.11 (Bolyen et al., 2019). Demultiplexed sequences were denoised with DADA2 (Callahan et al., 2016) (via q2‐dada2). Taxonomy was assigned to amplicon sequence variants (ASVs) using the q2‐feature‐classifier naïve Bayes taxonomy classifier (Bokulich et al., 2018) against the SILVA v138 rRNA gene database (Glöckner et al., 2017; Quast et al., 2013; Yilmaz et al., 2014). Metabarcode data were imported to R Core Team (2021; 4.1.0 2021‐05‐18) using the packages qiime2R (Bisanz, 2018), phyloseq (McMurdie & Holmes, 2013), and the tidyverse (Wickham et al., 2019). Alpha‐ and beta‐diversity analysis of metabarcoding data was performed on the relative abundance of 16S ASVs after samples were rarefied to a common depth (3155 reads per sample). Shannon–Wiener indices were calculated using the vegan package (Oksanen et al., 2020), and the difference between treatments was assessed by the Kruskal–Wallis rank‐sum test, followed by pairwise comparison with Dunn's test. Bray–Curtis distances were calculated with vegan (Oksanen et al., 2020), and visualized with ape (Paradis & Schliep, 2019) and differences between treatments were assessed through permutational multivariate ANOVA (999 permutations).

### 2.6 Plasmid extraction and bacterial transformation

The plasmid pUCITD‐AMP Golden Gate plasmid containing the synthetic erythromycin resistance gene (ermF) from IDT was used as a positive control for the transformation of DH5α cells. Bacterial plasmids were extracted using the QIAprep Spin Miniprep Kit, according to the manufacturer's instructions (Qiagen). Mix and Go chemically competent DH5α *Escherichia coli* cells were transformed with purified circular plasmids according to the manufacturer's instructions (Zymo Research) and colony PCRs for the *ermF* gene inserts were performed using forward: 5′‐TCGTTTTACGGGTCAGCACTTTAC‐3′ and reverse: 3′‐TTTCAGGGACAACTTCCAGCA‐5′ primers.

### 2.7 Disk diffusion assay

Disk diffusion assays were adapted from the EUCAST method (Matuschek et al., 2014), and bacteria were grown at 28°C overnight on Mueller–Hinton agar plates (Oxoid). Colonies of the isolate were selected and suspended in sterile saline (0.15 M) to the ideal concentration to create a turbidity comparison that matched 0.5 McFarland reagent (Thermo Scientific) (~1–2 × 10^8^ CFUs/mL). A sterile swab was submerged into the suspension and spread across Mueller–Hinton agar plates. Four disks containing antibiotics that included erythromycin (15 µg), gentamycin (10 µg), tetracycline (30 µg), and streptomycin (25 µg) as controls (Thermo Scientific) were applied to each Mueller–Hinton agar plate. The plates were incubated aerobically for 12–20 h at 28°C. Zones of inhibition were measured as the diameter of complete inhibition, which included the disk edge.

## 3 RESULTS

### 3.1 Manure

**Figure 2:**
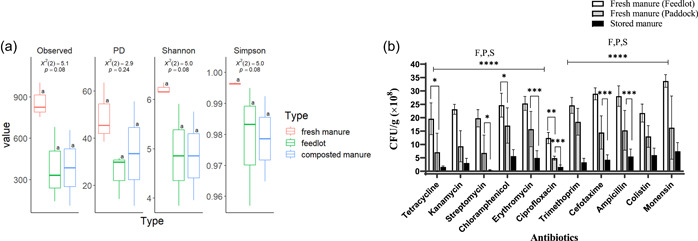
Bacterial diversity and antibiotic‐resistant bacteria (ARB) abundance in fresh and stored manure samples. (a) Alpha‐diversity analysis. (b) ARB colony‐forming units per gram (CFU/g). F, feedlot; P, paddock; PD, phylogenetic diversity; S, stored. ±SD error bars. *p < 0.05; **p < 0.01; ***p < 0.005; ****p < 0.0005.

Fresh paddock and feedlot manure, as well as composted manure from cattle treated prophylactically with monensin, were evaluated for microbial diversity and the abundance of ARB. As shown in Figure 2a, fresh paddock manure had higher bacterial alpha diversity compared to fresh feedlot and composted manure. Furthermore, Figure 2b showed an overall trend of increased ARB abundance in fresh paddock manure, compared to feedlot and composted manure. While the alpha diversity of feedlot and composted manure were similar, the composted manure had the lowest number of ARB observed, a trend that was consistent across all the tested antibiotics (Figure 2b). Unsurprisingly, the greatest number of ARB CFUs in the paddock, feedlot, and composted manure samples were observed for monensin, but also for trimethoprim, ampicillin, and cefotaxime. In contrast, the lowest ARB CFU was observed for ciprofloxacin (Figure 2b). Hence, fresh manure from the paddock had greater bacterial diversity but only intermediate levels of antibiotic resistance, suggesting that the increase in bacterial diversity is associated with a decrease in ARB abundance.

### 3.2 Bacterial and antibiotic‐resistant diversity in compost‐treated soil

**Figure 3:**
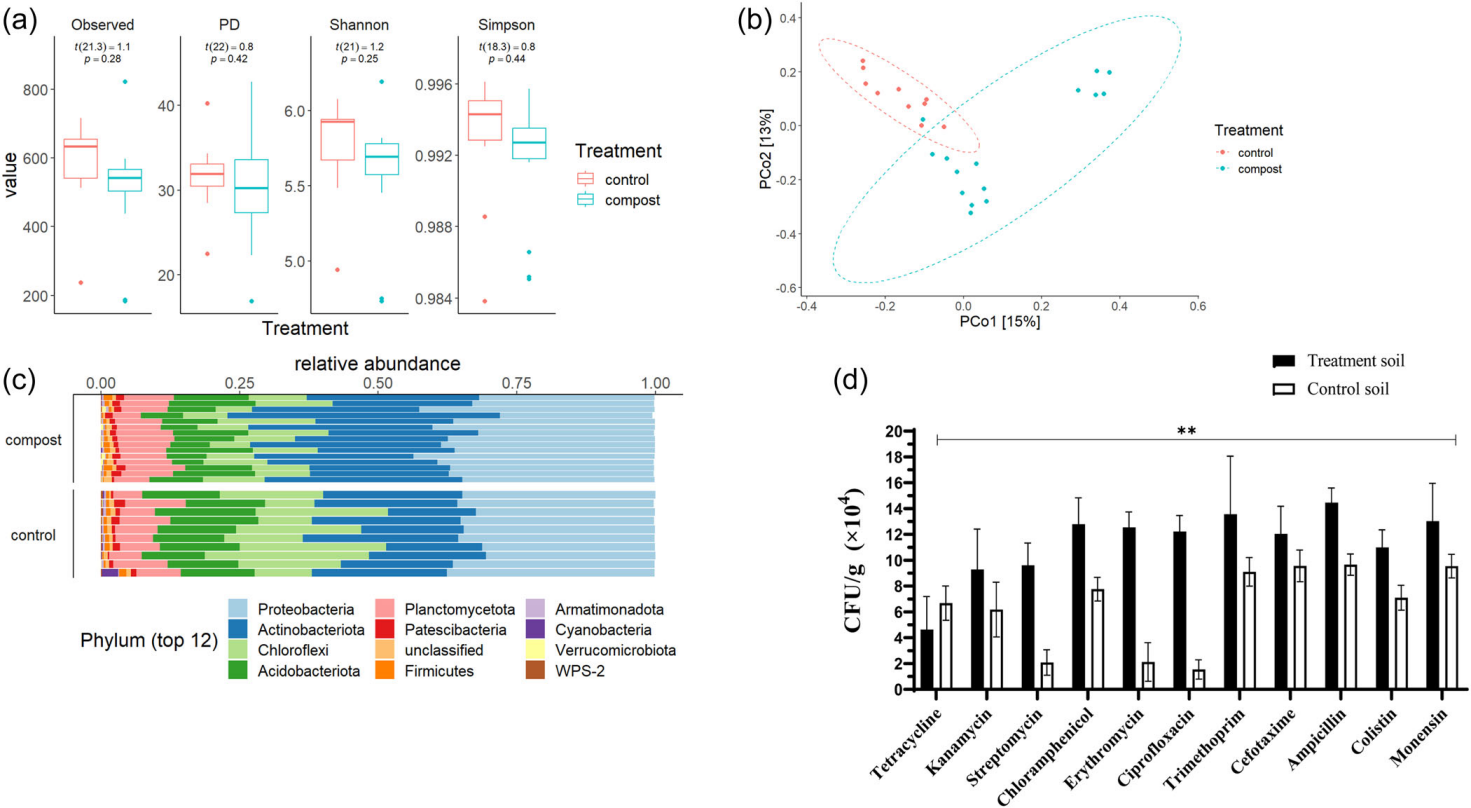
Bacterial diversity of soil samples, treated and untreated with composted manure. (a) Alpha‐diversity analysis using Shannon–Wiener indices. (b) Beta‐diversity analysis using Bray–Curtis distances matrix. (c) Relative phyla abundance in treated and control samples. (d) Evaluation of antibiotic‐resistant bacteria in soil, the number of bacterial colony‐forming units per gram (CFU/g) in compost‐treated (treatment) and untreated soils (control). PD, phylogenetic diversity. ±SD error bars. **p < 0.01.

To examine the effect of the composted manure application on paddock soils, the bacterial diversity and antibiotic resistance of compost‐treated and untreated control paddock soil were determined. There was no significant difference in alpha diversity (i.e., richness and evenness of species present) between untreated paddock soil and compost‐treated paddock soil (Figure 3a). However, beta‐diversity analysis (Figure 3b) demonstrated distinct bacterial community composition differences between the control, uncomposted paddock soil and the compost‐treated paddock soil. In addition, within the compost‐treated paddock, two discrete bacterial population clusters were present. Both the compost‐treated and control soil paddocks were dominated by Proteobacteria, Actinobacteriota, Chloroflexi, and Acidobacteriota phyla with differences in respective relative abundance means (relative atomic mass) of 0.34, 0.22, 0.18, and 0.14, respectively, for the control paddock soil and 0.36, 0.30, 0.1, and 0.1 in the compost‐treated paddock soil (Figure 3c).

A significantly (*p* < 0.01) greater abundance of ARB isolates was detected in the compost‐treated paddock soils compared to the control soils for all of the antibiotics examined (Figure 3d). In compost‐treated soils, high numbers of isolates had resistance to ampicillin (1.4 × 10^5^ CFU/g soil), trimethoprim (1.4 × 10^5^ CFU/g soil), monensin (1.3 × 10^5^ CFU/g soil), and ciprofloxacin (1.3 × 10^5^ CFU/g soil). By contrast, tetracycline had the lowest number of observed CFUs with 4.6 × 10^4^ CFU/g soil in both the compost‐treated and control soils, but resistance to tetracycline was higher in the control soil compared to the compost‐treated soil. (Figure 3d). Interestingly, samples from the control soil sites showed relatively high CFUs with resistance to the antibiotics: cefotaxime (1.0 × 10^5^ CFU/g soil), ampicillin (1.0 × 10^5^ CFU/g soil), and monensin (0.9 × 10^5^ CFU/g soil) (Figure 3d). Thus, the abundance of ARB in compost‐treated soil was significantly greater compared to control, untreated soil but the bacterial diversity structure of the compost‐treated and control bacterial soil communities was different.

### 3.3 16S identification of resistant isolates

**Figure 4:**
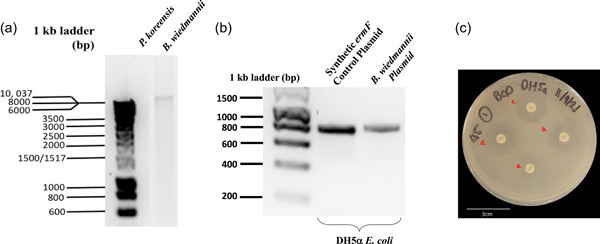
Isolates plasmid analysis. (a) Plasmid extractions from *Pseudomonas koreensis* and *Bacillus weidmanii*. (b) Colony PCR amplification of ermF gene fragment of the *B. wiedmanni* ARB plasmid isolates that was transformed into DH5α *Escherichia coli*. (c) Disk diffusion assay results for *B. wiedmanni* plasmid in DH5α transformants' antibiotic resistance screen using erythromycin (a.), gentamycin (b.), tetracycline (c.), and streptomycin (d.) impregnated disks.

The 16S sequence analysis was used to identify the ARB isolates from compost‐treated and control soils as well as from fresh manure to track diversity. The results found significant differences in the identity of resistant isolates analyzed from each group. At the phylum level, resistant manure isolates mostly belonged to the Proteobacteria phylum (74.2%) mainly of the genus *Pseudomonas*, followed by Bacillota (previously named Firmicutes, 12.9%), Actinomycetota (previously named Actinobacteria, 9.7%), and Bacteroidota (previously named Bacteroidetes, 3.2%). The resistant isolates from the soil microbial community largely belonged to the phyla Bacillota (57.1%) mainly from the genus *Bacillus*, and Proteobacteria (35.7%), followed by Actinomycetota (4.8%) and Bacteroidetes (2.4%). A complete list of the identified isolates and their resistance profiles is outlined in Appendix 1. Notably, the manure isolate *Pseudomonas* (accession number: NR_025228.1) and soil isolate *Bacillus* (accession number: NR_152692.1) demonstrated resistance against five different antibiotics. Because ARGs may be located on mobile genetic elements such as a plasmid, isolations from *Pseudomonas* (NR_025228.1) and *Bacillus* (NR_152692.1) isolates were performed. Figure 4 illustrated that a plasmid was successfully extracted from *Bacillus* (NR_152692.1). When this plasmid was transformed to antibiotic‐susceptible DH5α *E. coli*, it conferred antibiotic resistance activity against erythromycin but not to gentamycin, tetracycline, or streptomycin (Figure 4 and Table 2). The erythromycin resistance phenotype was further validated by sequence analysis demonstrating the plasmid carried a genetic element with 94% homology to the erythromycin resistance gene *ermF* (NG_047824.1) and 99.6% homology for the ermF protein sequence (AAF68230.1). Collectively, these data suggested that in *Bacillus* (NR_152692.1) erythromycin resistance was conferred by a mobile plasmid element, whereas the genes conferring resistance against gentamycin, tetracycline, or streptomycin remain unknown, but may be integrated into the genome of *Bacillus wiedmannii* FSL W8‐0169.

**Table 2:** Zones of inhibition for each antibiotic that was tested in the disk diffusion assay when confirming resistance to erythromycin in the transformed DH5α *Escherichia coli* cells.

	Erythromycin	Gentamycin	Tetracycline	Streptomycin
DH5α transformed with *ermF* plasmid	6	26	24.3	22
Control (DH5α *E. coli*)	12.33	20	24	21

## 4 DISCUSSION

### 4.1 ARB and microbial diversity in compost‐treated and untreated soils

The purpose of this study was to follow the development of antibiotic resistance from cattle to agricultural soils, via the application of different forms of animal manure as a soil treatment. Analysis of animal manure revealed a decreasing abundance of ARB from feedlot to paddock and finally stored manure, which contained low ARB abundance.

In agreement with a previous study, animal manure, particularly cattle manure, contains a substantial number of ARB even if the animal has not received antibiotics previously (Pu et al., 2019; Udikovic‐Kolic et al., 2014). Additionally, higher ARB loads have also been attributed to cattle in feedlots due to the accumulation of antibiotic residue in animal products (Chee‐Sanford et al., 2009; Heuer et al., 2011) as well as the limited space leading to increased transmission of ARB via direct contact among animals or through the ingestion of fecal contamination in feed and water sources (Netthisinghe et al., 2013). These conditions may act to apply selection pressure, thereby reducing the overall bacterial diversity of feedlot cattle compared to fresh manure from cattle grazed on paddocks, as has been observed here and in previous studies (Chang et al., 2021; Gaballah et al., 2021; Khan et al., 2008). However, composting manure significantly decreased the abundance and diversity of ARB mainly due to altered environmental conditions such as sunlight, temperature, pH values, and moisture, which all play a significant role in the degradation of antibiotics and the survival of the microorganisms (Chee‐Sanford et al., 2009; Koike et al., 2007; Martinez, 2009; Netthisinghe et al., 2013; Sengeløv, 2003). For these reasons, this study observed that the antibiotic resistance rates of bacteria in stored manure were very low compared to fresh manure from feedlot sites.

Spreading composted manure on paddocks produced divergent bacterial communities with a significantly higher abundance of ARB. This is in accordance with other observations in the literature (Pu et al., 2019; L. Wang et al., 2019). Considering that the compost treatment contained low amounts of live ARB, this raises the question, what is the resistance transfer mechanism, if it is not through the application of live ARB?

One hypothesis is that antibiotic resistance is transferred via resistant DNA or ARG mobile elements. Previous studies demonstrated that ARG and mobile genetic elements remain in the compost material following aerobic composting, long after the bacterial decay (Pu et al., 2019; Xie et al., 2016; Zhang et al., 2016). Once spread on the soil, ARG may disseminate and reach the soil microbial community, conferring antibiotic resistance through horizontal gene transfer (Pu et al., 2019). Analysis of the common resistant isolates identified in this study revealed a novel plasmid carrying the erythromycin resistance gene, with demonstrated capacity to confer resistance to erythromycin in other microbes from different genera. Further analysis should be aimed at characterizing antibiotic resistance‐carrying mobile genetic elements that are present in compost.

Another mechanism could be due to antibiotic residues within the animal manure that are introduced into the soil with compost application, thus contributing to the accumulation of antibiotics in the soil (Chang et al., 2015; Speicher, 2016), and creating selection pressure that leads to the observed increase in ARB. Previous studies have found that significant concentrations of antibiotics are accumulated in commercial compost of animal manure (J. Wang et al., 2015; Xie et al., 2016). Further studies are needed to understand the mechanisms of antibiotic resistance transfer from compost to the soil and to develop strategies to minimize this process. Interestingly, compost application not only acted to increase ARB abundance but also influenced the overall diversity of the soil microbial population. Further research is needed to identify which components in the compost are the main facilitators of this effect.

### 4.2 Effect of compost treatment on the development of specific antibiotic resistance

The highest number of ARB in manure samples from feedlot and compost samples was for monensin (Figure 2). This is not surprising as monensin was given prophylactically to the cattle. Following monensin, high resistance was observed for the antibiotics, cefotaxime, and ampicillin, which inhibit cell wall synthesis (Figure 2). This is consistent with previous investigations that reported how bacterial isolates from cattle manure such as *Salmonella* spp., *E. coli*, and *Pseudomonas* spp. showed high resistance levels for ampicillin, cefotaxime, and monensin (Camiade et al., 2020; Ibrahim et al., 2016; Xu et al., 2018).

Interestingly, we detected a high abundance of ARBs in the untreated soil. In the case of tetracycline, ARB abundance in the untreated soil was higher than the one observed in the treated soil (Figure 3). This high resistance may be a result of historical antibiotic use, before the documented history of these soils; alternatively, this soil could be naturally high in tetracycline‐resistant bacteria whose abundance was decreased in the treated soils as a result of continuing compost application.

## 5 CONCLUSION

In conclusion, the current study demonstrates that the application of cattle manure compost as a soil treatment acts to increase the abundance of antimicrobial resistance in soil and reduce the microbial diversity of the soil. This contributes to the global increase in antibiotic resistance, which has extended implications on both animal and human health, as defined by the one health framework. This study highlights the importance of understanding the transfer mechanism for antibiotic resistance from manure to the soil to maintain soil fertilization without enhancing antibiotic resistance.

## AUTHOR CONTRIBUTIONS

**Fadhel Abbas**: Conceptualization (supporting); data curation (lead); methodology (equal); project administration (equal). **Phil Thomas**: Data curation (equal); formal analysis (equal); methodology (equal); project administration (equal). **Bianca Cully‐Duse**: Data curation (supporting); formal analysis (supporting); methodology (equal). **Nicholas M. Andronicos**: Conceptualization (lead); data curation (equal); formal analysis (equal); methodology (lead); project administration (equal). **Gal Winter**: Conceptualization (lead); data curation (supporting); formal analysis (lead); methodology (lead); project administration (equal).

## CONFLICT OF INTEREST STATEMENT

None declared.

## ETHICS STATEMENT

None required.

## Data Availability

All data are provided in full in this paper except for 16S rRNA diversity sequencing data, which are available at the Research UNE repository (University of New England): https://doi.org/10.25952/6kth-k110.

## APPENDIX 1

Table A1

**Table A1:** A complete list of the identified isolates and their resistance profiles.

Bacterial isolates	Phylum	Number	Gram stain	Source of isolate	Antibiotic resistance										
					Tetracycline	Trimethoprim	Cefotaxime	Ampicillin	Erythromycin	Kanamycin	Streptomycin	Ciprofloxacin	Chloramphenicol	Colistin	Monensin
*Bacillus wiedmannii*	Firmicutes	9	Positive	Soil					x	x	x	x	x		
*Acinetobacter pittii*	Proteobacteria	5	Negative	Soil	x	x			x				x		
*Bacillus paranthracis*	Firmicutes	3	Positive	Soil			x			x					
*Bacillus pacificus*	Firmicutes	3	Positive	Soil			x	x			x				
*Acinetobacter lwoffii*	Proteobacteria	2	Negative	Soil				x			x				
*Pseudomonas punonensis*	Proteobacteria	2	Negative	Soil	x	x									
*Bacillus paramycoides*	Firmicutes	2	Positive	Soil									x	x	
*Bacillus cereus*	Firmicutes	2	Positive	Soil		x									
*Enterobacter cloacae*	Proteobacteria	2	Negative	Soil		x									
*Pseudomonas putida*	Proteobacteria	1	Negative	Soil		x									
*Pseudomonas mediterranea*	Proteobacteria	1	Negative	Soil									x	x	
*Pseudomonas koreensis*	Proteobacteria	1	Negative	Soil					x						
*Bacillus mobilis*	Firmicutes	1	Positive	Soil										x	
*Bacillus zanthoxyli*	Firmicutes	1	Positive	Soil									x		
*Bacillus pseudomycoides*	Firmicutes	1	Positive	Soil	x										
*Pseudoxanthomonas sacheonensis*	Proteobacteria	1	Negative	Soil				x					x		
*Lysinibacillus mangiferihumi*	Firmicutes	1	Positive	Soil				x							
*Chryseobacterium aquifrigidense*	Bacteroidetes	1	Negative	Soil					x						
*Paenarthrobacter nitroguajacolicus*	Actinomycetota	1	Positive	Soil								x			
*Paenarthrobacter aurescens*	Actinomycetota	1	Positive	Soil									x		
*Paenibacillus lautus*	Firmicutes	1	Positive	Soil							x				
*Pseudomonas koreensis*	Proteobacteria	6	Negative	Manure		x	x		x		x			x	
*Bacillus wiedmannii*	Firmicutes	3	Positive	Manure				x		x					x
*Pseudomonas marginalis*	Proteobacteria	2	Negative	Manure		x				x					
*Arthrobacter koreensis*	Actinomycetota	1	Positive	Manure				x							
*Arthrobacter luteolus*	Actinomycetota	1	Positive	Manure										x	
*Alkanindiges hongkongensis*	Proteobacteria	1	Negative	Manure	x										
*Proteus vulgaris*	Proteobacteria	1	Negative	Manure							x				
*Sphingobacterium faecium*	Bacteroidetes	1	Negative	Manure									x		
*Aeromonas rivuli*	Proteobacteria	1	Negative	Manure								x			
*Enterobacter tabaci*	Proteobacteria	1	Negative	Manure									x		
*Psychrobacter pulmonis*	Proteobacteria	1	Negative	Manure					x						
*Psychrobacter faecalis*	Proteobacteria	1	Negative	Manure										x	
*Solibacillus kalamii*	Firmicutes	1	Positive	Manure	x										
*Glutamicibacter arilaitensis*	Actinomycetota	1	Positive	Manure							x				
*Robbsia andropogonis*	Proteobacteria	1	Positive	Manure											x
*Acinetobacter calcoaceticus*	Proteobacteria	1	Negative	Manure											
*Pseudomonas brenneri*	Proteobacteria	1	Negative	Manure	x										
*Pseudomonas migulae*	Proteobacteria	1	Negative	Manure		x									
Pseudomonas moraviensis	Proteobacteria	1	Negative	Manure		x									
*Pseudomonas veronii*	Proteobacteria	1	Negative	Manure			x								
*Pseudomonas canadensis*	Proteobacteria	1	Negative	Manure					x						
*Pseudomonas songnenensis*	Proteobacteria	1	Negative	Manure			x								
*Pseudomonas extremaustralis*	Proteobacteria	1	Negative	Manure				x							
